# Quantitative modeling of multigenerational effects of chronic ionizing radiation using targeted and nontargeted effects

**DOI:** 10.1038/s41598-021-84156-2

**Published:** 2021-02-26

**Authors:** Igor Shuryak, David J. Brenner

**Affiliations:** Center for Radiological Research, Columbia University Irving Medical Center, 630 West 168th Street, VC-11-234/5, New York, NY 10032 USA

**Keywords:** Biophysics, Computational biology and bioinformatics, Risk factors

## Abstract

Stress response signals can propagate between cells damaged by targeted effects (TE) of ionizing radiation (e.g. energy depositions and ionizations in the nucleus) and undamaged “bystander” cells, sometimes over long distances. Their consequences, called non-targeted effects (NTE), can substantially contribute to radiation-induced damage (e.g. cell death, genomic instability, carcinogenesis), particularly at low doses/dose rates (e.g. space exploration, some occupational and accidental exposures). In addition to controlled laboratory experiments, analysis of observational data on wild animal and plant populations from areas contaminated by radionuclides can enhance our understanding of radiation responses because such data span wide ranges of dose rates applied over many generations. Here we used a mechanistically-motivated mathematical model of TE and NTE to analyze published embryonic mortality data for plants (*Arabidopsis thaliana*) and rodents (*Clethrionomys glareolus*) from the Chernobyl nuclear power plant accident region. Although these species differed strongly in intrinsic radiosensitivities and post-accident radiation exposure magnitudes, model-based analysis suggested that NTE rather than TE dominated the responses of both organisms to protracted low-dose-rate irradiation. TE were predicted to become dominant only above the highest dose rates in the data. These results support the concept of NTE involvement in radiation-induced health risks from chronic radiation exposures.

## Introduction

Over the first several decades after the discovery of biological effects of ionizing radiation, these effects were interpreted mainly by target theory, which postulates that radiation damage (e.g. cell death, mutations) is caused by energy depositions and ionizations within sensitive cellular targets. Radiobiological research led towards identification of genomic DNA as the main target^1^ and the double strand break (DSB) as the most important severe radiation-induced DNA lesion^2, 3^. Implicitly, target theory assumed that cells in a multicellular organism are effectively independent from each other with respect to radiation-induced damage, so that no such damage was expected to occur in cells that were not “hit” by radiation tracks.

Some evidence that did not fit into this paradigm, where radiation apparently affected those cells or organisms that were not directly exposed, but interacted with exposed counterparts in some manner, gradually accumulated over several decades (reviewed by^4–9^). Main-stream radiobiological attention was drawn to such phenomena, which were later called bystander effects (BE) or non-targeted effects (NTE), after the study by Nagasawa and Little^10^. This work showed that in cells exposed to ^238^Pu alpha particles, the dose response for sister chromatid exchanges rose steeply in the dose range where most cell nuclei were not expected to be “hit” by any particle tracks.

NTE were subsequently detected by many laboratories (reviewed by^4, 5, 9, 11–13^), using different ionizing radiation types—e.g. high linear energy transfer (LET) particles as well as low-LET photons—in a wide variety of biological systems including cells and whole organisms. NTE were demonstrated not only in mammalian cell cultures and laboratory mammals such as mice, but also in fish^14, 15^, invertebrate animals (e.g.* Caenorhabditis elegans*)^16, 17^ and in plants (e.g.* Arabidopsis thaliana*)^18, 19^. NTE encompass a very wide range of biological outcomes, including cell death, mutagenesis, oncogenic transformation, micronucleus formation, genomic instability, gene expression changes, senescence, migration and/or differentiation alterations (reviewed by^4, 5, 9, 11–13, 20^). Similar effects could be induced not only by ionizing radiation, but also by heavy metals, chemotherapy agents and other toxic chemicals, and by photodynamic stress^4, 11, 21^.

The common theme underlying NTE appears to be propagation of stress responses between cells within an organism, potentially through the germline to subsequent generations, or even between different organisms^14, 22^. This phenomenon relies on many short-range and long-range signaling mechanisms and probably has a very ancient evolutionary origin, predating the appearance of multicellular organisms. For example, unicellular bacteria can coordinate their stress responses by quorum sensing^23, 24^. Such mechanisms of intercellular communication likely evolved to respond to natural stressors such as toxic chemicals or infections. Difficult to repair complex DNA lesions, such as clusters of DNA double strand breaks in close proximity to one another, potentially with accompanying single strand breaks and base damage, represent an important type of radiation damage which can induce persistent NTE signal release and other adverse outcomes^25–30^. Sometimes they are protective against ionizing radiation exposure (e.g. adaptive responses, terminal cell differentiation), but in other cases “overreactive” and persistent stress responses cause harmful outcomes (e.g. genomic instability, chronic inflammation).

One of the most important deleterious NTE endpoints is genomic instability: persistently elevated rate of mutations and/or genomic rearrangements not only in directly irradiated cells, but also in bystander cells that interacted with the irradiated cells, and in their descendants^11^. This phenomenon, which was particularly well studied in rodents and in some invertebrate animals such as *Daphnia*, is most likely mediated by epigenetic mechanisms and can be transmitted across generations^31–35^. Transgenerational genome destabilization can be caused not only by ionizing radiation, but also by chemical mutagens^34^.

**Figure 1:**
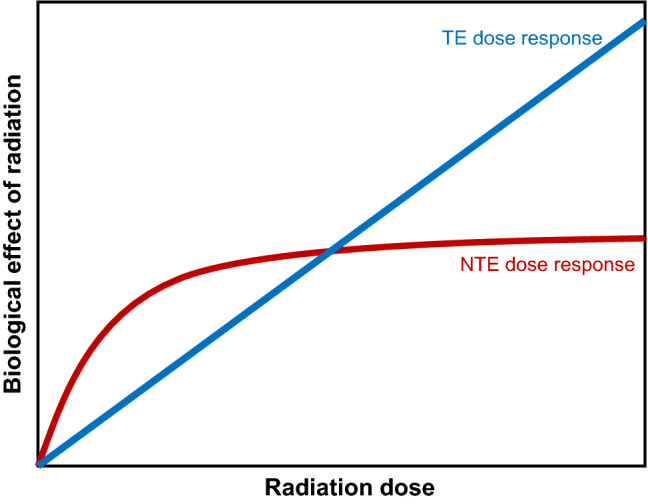
Schematic comparison of typical radiation dose response shapes for targeted effects (TE) and non-targeted effects (NTE).

Importantly, NTE tend to qualitatively differ from TE in terms of radiation dose response shapes, as shown schematically in Fig. 1. TE tend to have linear or upwardly-curving linear-quadratic dose responses (with a positive second derivative). They are usually interpreted as accumulation of energy deposition events from single radiation tracks (linear dose response component) and multiple interacting tracks (approximated by the quadratic component)^36–38^. In contrast, NTE dose response shapes tend to be concave functions (with a negative second derivative) that deviate from zero very quickly at low doses and saturate/plateau at higher doses^5, 10, 11, 39^. These shapes are intuitively plausible, considering that NTE are caused by onset and perpetuation of a stressed state by signaling pathways in large groups of cells responding to damage initially induced in a small proportion of “hit” cells. The behavior of this system can resemble a binary on/off phenomenon, where the probability of the “on” switch increases with dose, but the magnitude of the effect is constant. Due to these properties, NTE can substantially contribute to radiation-induced damage, particularly at low doses/dose rates (e.g. space exploration, some occupational and accidental exposures).

The presence of a concave/saturating dose response component at low radiation doses, where statistically not all cell nuclei are expected to be “hit” over a relevant time period such as a cell cycle, can represent circumstantial evidence for NTE involvement. This conclusion was involved in the analysis of various data sets, e.g. human and rodent lung cancers induced by radon exposure^39–41^, mouse tumors induced by high-LET radiations^42–44^, chromosomal aberration and liver tumor yields in hamsters injected with plutonium isotopes (reviewed in^4^), chromosomal aberrations in snail embryos^45^ and embryonic mortality in wild rodents in areas contaminated by fallout from the Chernobyl accident^46^.

The latter type of data sets, generated by observational studies of wild animal and plant populations from areas contaminated by radionuclides released by nuclear accidents such as Chernobyl and Fukushima, have several strengths and weaknesses, relative to controlled laboratory experiments. For example, observational studies conducted under field conditions can suffer from limitations in the accuracy of radiation dosimetry for studied organisms (e.g. because concentrations of radionuclides incorporated into plants and animals can vary widely even between individuals of the same species collected from the same location), and not all potentially important variables that can affect the studied response (e.g. variations in temperature and rainfall, intra- and inter-specific competition, and changes in land use patterns after abandonment of contaminated areas by humans) can be identified and accounted for^47^. However, field studies from radioactively contaminated regions can be uniquely valuable because they produce data that span wide ranges of dose rates and radiation types (e.g. mixtures of gamma, beta and alpha-emitting radionuclides) applied over many generations. Analysis of such data, along with those from laboratory experiments, can enhance our understanding of ionizing radiation effects, including NTE.

Here we used a mechanistically-motivated mathematical model of TE and NTE, based on our previous work^41, 44, 45, 48, 49^, to analyze published embryonic mortality data for plants (Arabidopsis, *Arabidopsis thaliana*)^50^ and rodents (bank vole, *Myodes* or *Clethrionomys glareolus*) from the Chernobyl nuclear power plant accident region^51^. These data are valuable because they were collected relatively soon after the disaster (starting within 1–2 years after the accident in 1986), when radiation dose rates were highest and their effects were likely to be most prominent, and covered many generations of studied plants and animals, with each subsequent generation being exposed to a lower dose rate due to radionuclide decay and ecological processes. Our goal in analyzing such data was to investigate the model-predicted roles of NTE versus TE as function of radiation dose rate and time since the start of exposure, and to compare these results across species. We believe that such studies provide potentially useful evidence about the mechanisms of radiation-induced health risks at low doses/dose rates.

## Materials and methods

### Mathematical model

We previously developed a simple mathematical model of radiation-induced NTE and implemented it on several human and animal data sets^41, 44, 45, 48, 49^. Although the signaling pathways involved in NTE are complex and incompletely understood, their consequences can be quantitatively modeled by using the following set of assumptions: (1) irradiated cells “activate” other cells in an “on–off” (binary) manner by NTE signals, causing them to enter into a prolonged stressed state, which can be transmitted across generations (e.g. epigenetically). (2) “Activated” cells accumulate damage at an elevated rate. (3) Eventually they can revert to the background state, but this process may be very slow. (4) NTE-induced damage adds to the damage produced by direct traversal of targets by radiation (TE).

The adaptation of this modeling approach to the data sets on embryonic mortality during multi-generational exposure to chronic radiation, where the dose rate decreases over time, is described by the following system of ordinary differential equations. Here *R* is the radiation dose rate, *t* is time after the start of irradiation, *P*_*a*_ is the average probability of cells to be in an "activated" state due to NTE signals induced by radiation, and *Y* is radiation-induced yield of the damage of interest (embryonic mortality in this case).

1 $$\frac{d{P}_{a}\left(t\right)}{dt}={k}_{1}\times R\left(t\right)\times \left(1-{P}_{a}\left(t\right)\right)-{c}_{3}\times {P}_{a}\left(t\right)$$

2 $$\frac{dY\left(t\right)}{dt}={k}_{bac}+{k}_{TE}\times R\left(t\right)+{k}_{NTE}\times {P}_{a}\left(t\right)-\kappa \times Y\left(t\right).$$

The parameters *k*_1_ (dose^−1^) and *c*_3_ (time^−1^) represent cell “activation” and deactivation by NTE, respectively. This notation is the same as in our original papers on NTE modeling^48, 49^. The parameter *k*_*bac*_ (time^−1^) represents background *Y* formation, *k*_*TE*_ (dose^−1^) represents *Y* formation by TE, *k*_*NTE*_ (time^−1^) represents the proportionality constant relating *P*_*a*_ to *Y*, and κ (time^−1^) represents *Y* removal (e.g. due to damage repair and selection against heavily damaged cells/organisms in the population).

Without radiation exposure (*R*(*t*) = 0), the system (Eqs. 1, 2) approaches an equilibrium condition where *P*_*a*_(*t*) = 0 and *Y*(*t*) = *k*_*bac*_/κ. For irradiation at constant dose rate (*R*(*t*) = *R*_*c*_), the system can be solved analytically as follows:

3 $${P}_{a}\left(t\right)=-\frac{\left[{k}_{1}\times {R}_{c}\times \left(\mathrm{exp}\left[{-X}_{1}\times t\right]-1\right)\right]}{{X}_{1}}$$

4 $$Y\left(t\right)=\frac{{k}_{NTE}\times {k}_{1}\times\upkappa \times {R}_{c}\times \mathrm{exp}\left[-{X}_{1}\times \mathrm{t}\right]-{X}_{1}\times {X}_{2}\times {R}_{c}\times \mathrm{exp}\left[-\upkappa \times \mathrm{t}\right]+{X}_{3}}{\left[\upkappa \times {X}_{1}\times \left({X}_{1}-\upkappa \right)\right]}$$

5 $${X}_{1}={k}_{1}\times {R}_{c}+{c}_{3}$$

6 $${X}_{2}={k}_{1}\times {k}_{TE}\times {R}_{c}+{k}_{NTE}\times {k}_{1}+{c}_{3}\times {k}_{TE}-{k}_{TE}\times\upkappa$$

7 $${X}_{3}=\left[{k}_{1}\times {k}_{TE}\times {R}_{c}^{2}+\left(\left[{k}_{NTE}+{k}_{bac}\right]\times {k}_{1}+{c}_{3}\times {k}_{TE}\right)\times {R}_{c}+{k}_{bac}\times {c}_{3}\right]\times \left[{X}_{1}-\upkappa \right].$$

Assuming that all model parameters are greater than zero, after long times at constant dose rate the system tends towards the following equilibrium solution:

8 $${P}_{a}\left(t\right)={k}_{1}\times \frac{{R}_{c}}{{X}_{1}}, Y\left(t\right)={X}_{3}/[\upkappa \times {X}_{1}\times ({X}_{1}-\upkappa )].$$

However, here we are interested in the situation where radiation dose rate is not constant, but decreases exponentially over time since the Chernobyl nuclear accident according to the following equation, where *R*_0_ is the initial dose rate right after the accident and λ is its rate of decrease:

9 $$R\left(t\right)={R}_{0}\times \mathrm{exp}\left[-\lambda \times t\right].$$

Of course, this exponential time dependence for the dose rate is only a rough approximation for environmental exposures after an accident because complex processes of radionuclide migration and bioaccumulation in different soil layers and biomass of different organisms are not explicitly accounted for. However, the exponential dependence does account for the main trend in dose rate after an accident with the minimal number of adjustable parameters.

When the exponential dose rate dependence on time (Eq. 9) was substituted into the system of model equations (Eqs. 1, 2), we could no longer find an analytic solution and solved the system numerically using the *dsolve numeric* procedure in Maple 2019 software. Since *Y*(*t*) in our model represents the mean number of lethal events (a continuous number > 0), whereas the observed data are probabilities of embryonic mortality (bounded between 0 and 1), we used the following formula based on an assumed Poisson distribution of lethal events to generate model predictions for the probability of embryonic mortality (*P*_*mort*_), which is the probability of observing ≥ 1 lethal event:

10 $${P}_{mort}=1-\mathrm{exp}[-Y\left(t\right)]$$

This approach was used to fit predicted *P*_*mort*_ values to the plant and rodent data sets, as described below.

### Data sets

The plant (*Arabidopsis thaliana*) data set was obtained from Tables 1–8 in the publication by Abramov et al.^50^. Three variables were used for analysis: (1) time since the beginning of irradiation (since the Chernobyl accident in this case), (2) radiation dose rate at each time point at each studied location, and (3) the corresponding embryonic mortality yield at each time point at each studied location.

The radiation dose rate at each of 14 locations (Chernobyl, Shepelichi, Stechanka, Tolstyi Les, Yanov-0, Yanov-1, Yanov-2, Yanov-3, Yanov-4, Yanov-6, Yanov-7, Yanov-8, Yanov-10, Damba) was calculated based on the “monthly dose” for the sum of γ and β radiations reported by Abramov et al*.*^50^, converted to units of µGy/h (assuming 1 month = 30 × 24 h). Dose rate was ln-transformed to bring the error distribution closer to Normal. Robust linear regression with time since 1986 (in years) as the independent variable, and ln-transformed dose rate (in µGy/h) as the dependent variable, was performed (using the *rlm* function in *R* 4.0.2 software) separately for each location. This regression generated location-specific estimates of the dose rate at time zero (parameter *R*_0_) and the exponential rate of decrease for the dose rate (λ, years^−1^). For those locations where there were not enough time measurements to perform the regression (e.g*.* dose rate measured only at one time), λ was set to approximately zero (10^–3^ years^−1^, to avoid a singularity in solving the model equations) and *R*_0_ was set to the mean of available dose rate measurements. The robust procedure was selected instead of ordinary least squares regression to minimize the potential effect of “outlier” data points on the *R*_0_ and λ estimates. The embryonic mortality yield was based on the “Chimeras for lethals, %” values reported by Abramov et al*.*^50^, with the addition of “the maximum frequency observed in control plants (5%)”. The resulting data set composed of 35 data points is provided in Table 1.

**Table 1:** Comparison of observed and fitted embryonic mortality in the plant data set.

Location	Time since 1986 (years)	Dose rate (µGy/h)	Robust linear regression fit to ln[dose rate] as function of time		Observed embryonic mortality	Fitted embryonic mortality
			Intercept (R_0_)	Slope (λ, years^−1^)		
Chernobyl	1	73.6	4.56	0.28	0.070	0.119
Chernobyl	2	52.5	4.56	0.28	0.172	0.141
Chernobyl	3	42.0	4.56	0.28	0.227	0.153
Chernobyl	4	31.5	4.56	0.28	0.196	0.159
Chernobyl	5	21.0	4.56	0.28	0.166	0.162
Chernobyl	6	10.5	4.56	0.28	0.107	0.162
Shepelichi	1	1224.0	6.74	0.09	0.154	0.122
Shepelichi	2	720.0	6.74	0.09	0.224	0.146
Shepelichi	3	648.1	6.74	0.09	0.218	0.159
Shepelichi	4	576.0	6.74	0.09	0.167	0.167
Shepelichi	6	504.0	6.74	0.09	0.116	0.175
Stechanka	1	2.1	0.74	0.00	0.066	0.094
Stechanka	2	2.1	0.74	0.00	0.100	0.102
Stechanka	3	2.1	0.74	0.00	0.221	0.107
Stechanka	4	2.1	0.74	0.00	0.062	0.110
Stechanka	6	2.1	0.74	0.00	0.074	0.113
Tolstyi Les	1	322.5	6.05	0.26	0.078	0.121
Tolstyi Les	2	258.1	6.05	0.26	0.090	0.144
Tolstyi Les	3	193.5	6.05	0.26	0.138	0.157
Tolstyi Les	4	129.0	6.05	0.26	0.148	0.165
Tolstyi Les	6	90.3	6.05	0.26	0.139	0.171
Yanov-6	2	23,919.4	11.13	0.50	0.200	0.230
Yanov-6	3	16,560.0	11.13	0.50	0.283	0.235
Yanov-6	4	9200.0	11.13	0.50	0.250	0.228
Yanov-6	5	5520.0	11.13	0.50	0.247	0.217
Yanov-8	3	875.0	6.77	0.00	0.205	0.160
Yanov-8	4	875.0	6.77	0.00	0.111	0.168
Yanov-0	1	1288.1	7.16	0.00	0.125	0.123
Yanov-1	1	2208.3	7.70	0.00	0.104	0.124
Yanov-2	1	4415.3	8.39	0.00	0.180	0.128
Yanov-3	1	23,919.4	10.08	0.00	0.131	0.156
Yanov-4	1	44,159.7	10.70	0.00	0.141	0.184
Yanov-7	2	26,680.6	10.19	0.00	0.216	0.203
Damba	5	52.5	3.96	0.00	0.166	0.164
Yanov-10	5	87.5	4.47	0.00	0.152	0.167

R^2^ for observed versus model-fitted values was 0.43, and RMSE was 0.044.

The rodent (*Clethrionomys glareolus*) data set was obtained from Table 3 in the publication by Ryabokon et al*.*^51^. The radiation dose rate at each of 3 locations (called sites 2, 3 and 4) was calculated based on the “Whole-body absorbed dose rate” for the sum of γ and β radiations reported by Ryabokon et al*.*^51^, converted from µGy/day to µGy/h. For two instances of background conditions where no dose rate value was reported by Ryabokon et al*.*^51^, we assumed 0.05 µGy/day = 0.00208 µGy/h.

As for the plant data described above, dose rate was ln-transformed to bring the error distribution closer to Normal. Robust linear regression was performed separately for each location to estimate parameters *R*_0_ and λ. For those locations where there were not enough time measurements to perform the regression, λ was set to 10^–3^ years^−1^ and *R*_0_ was set to the mean of available dose rate measurements, for the reasons described above. The embryonic mortality yield was based on the “Mean embryonic lethality” values (totals for before and after implantation) reported by Ryabokon et al*.*^51^. The resulting data set composed of 12 data points is provided in Table 2.

**Table 2:** Comparison of observed and fitted embryonic mortality in the rodent data set.

Location	Time since 1986 (years)	Dose rate (µGy/h)	Robust linear regression fit to ln[dose rate] as function of time		Observed embryonic mortality	Fitted embryonic mortality
			Intercept (R_0_)	Slope (λ, years^−1^)		
Site 2	0	0.0021	− 6.17	0.00	0.072	0.022
Site 2	6	0.0021	− 6.17	0.00	0.044	0.022
Site 2	5	0.23	− 0.69	0.16	0.054	0.054
Site 2	10	0.10	− 0.69	0.16	0.114	0.112
Site 3	2	2.42	1.36	0.28	0.000	0.051
Site 3	3	1.85	1.36	0.28	0.065	0.072
Site 3	5	0.75	1.36	0.28	0.099	0.115
Site 3	10	0.27	1.36	0.28	0.267	0.218
Site 4	2	10.23	2.89	0.24	0.048	0.066
Site 4	3	11.07	2.89	0.24	0.060	0.089
Site 4	5	4.32	2.89	0.24	0.155	0.134
Site 4	10	1.81	2.89	0.24	0.216	0.236

R^2^ for observed versus model-fitted values was 0.84, and RMSE was 0.029.

Both *Arabidopsis thaliana* and *Clethrionomys glareolus* have short generation times, generally less than 1 year, and sometimes 2 per year, under the studied conditions^50, 51^. Therefore, these data sets analyzed here extend to multiple generations of each species.

### Model fitting procedure

The model equation for embryonic mortality yield under an exponentially decreasing dose rate (Eq. 9 substituted into Eq. 2) was fitted separately to each data set (plant and rodent) using a customized iterative algorithm implemented in maple 2019 software. This algorithm is described in Supplementary Methods online. Uncertainties of the best-fit model parameters on each data set were estimated by generating 1000 randomly perturbed versions of each data set by bootstrapping with replacement. The fitting procedure was applied to each perturbed data set, starting with the best-fit parameter combination previously found on the original (not perturbed) version of the corresponding data set. The 2.5th and 97.5th percentiles of the distribution of best-fit values of each parameter over the 1000 perturbed data sets were used as estimates of the 95% confidence interval (CI) of the selected parameter.

## Results

**Figure 2:**
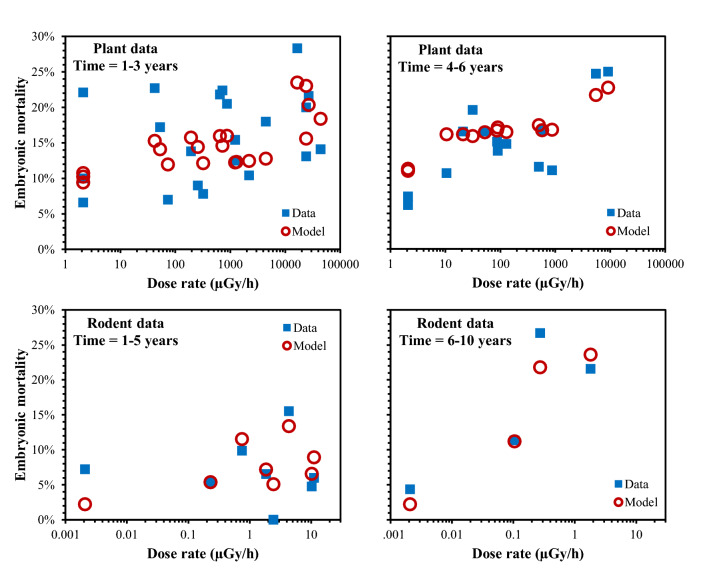
Comparison of observed data for plants (*Arabidopsis thaliana*) and rodents (*Clethrionomys glareolus*) with best-fit model predictions, as function of radiation dose rate and time after the Chernobyl accident. Model predictions are shown as symbols rather than as continuous curves because they do not change monotonically as function of dose rate measured at a given time after the accident, since they correspond to data from different locations with different dependences of dose rate on time (see Tables 1, 2). Continuous curves based on model predictions, which improve visualization of model behaviors, are shown in subsequent figures.

**Figure 3:**
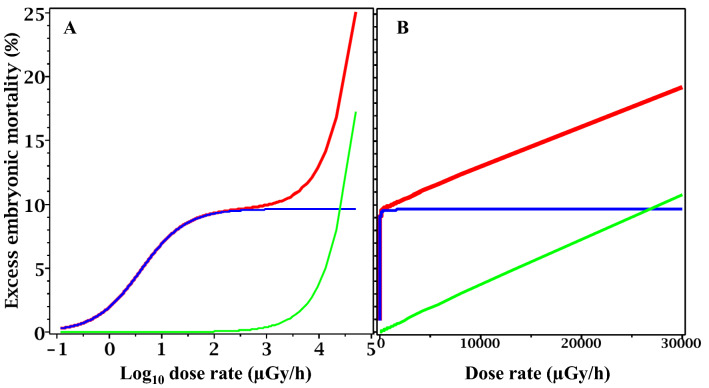
Predicted radiation responses at different constant dose rates based on best-fit model parameters for plant (*Arabidopsis thaliana*) data. (**A**) Dose rate shown on logarithmic scale for better visualization of low dose rates. (**B**) dose rate shown on linear scale. Red curve = total radiation response (TE and NTE). Blue curve = NTE only. Green curve = TE only. Time since the start of exposure to the constant dose rate was set to 20 years so that near-equilibrium for the response could be achieved. In this and the following figures, background embryonic mortality was subtracted for better visualization of the radiation responses.

**Figure 4:**
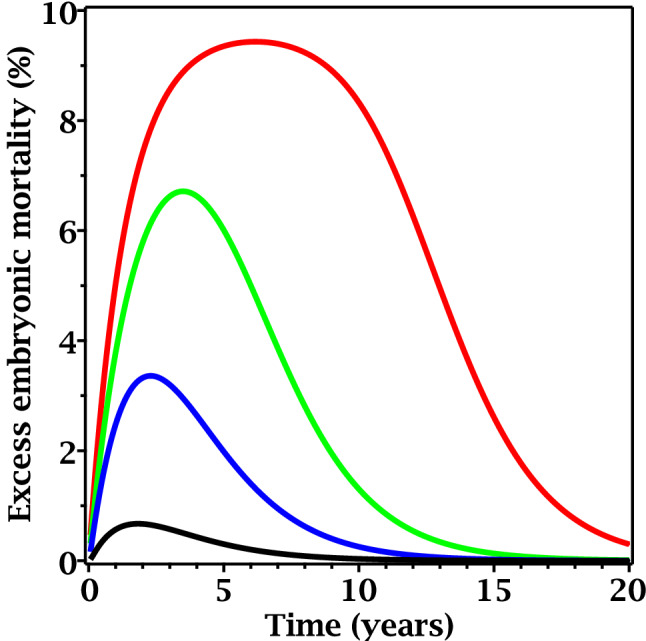
Predicted radiation responses as function of time since the start of exposure to different time-dependent dose rates based on best-fit model parameters for plant (*Arabidopsis thaliana*) data. In all curves, the time needed for dose rate to decrease by twofold is 1 year. Red curve: initial dose rate = 10,000 µGy/h. Green curve: initial dose rate = 100 µGy/h. Blue curve: initial dose rate = 10 µGy/h. Black curve: initial dose rate = 1 µGy/h.

**Figure 5:**
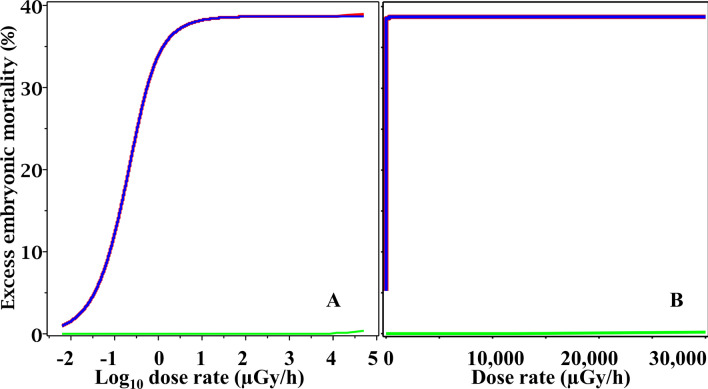
Predicted radiation responses at different constant dose rates based on best-fit model parameters for rodent (*Clethrionomys glareolus*) data. (**A**) Dose rate shown on logarithmic scale for better visualization of low dose rates. (**B**) Dose rate shown on linear scale. Red curve = total radiation response (TE and NTE). Blue curve = NTE only. Green curve = TE only. Time since the start of exposure to the constant dose rate was set to 20 years so that near-equilibrium for the response could be achieved.

**Figure 6:**
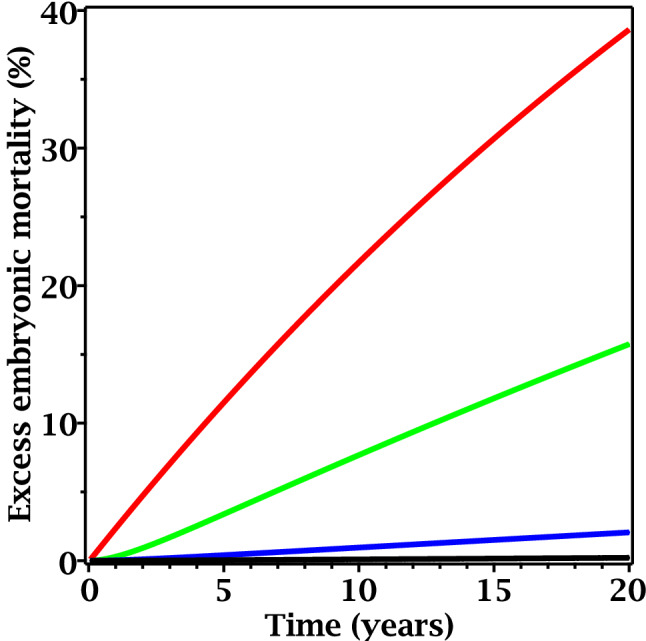
Predicted radiation responses as function of time since the start of exposure to different time-dependent dose rates based on best-fit model parameters for rodent (*Clethrionomys glareolus*) data. In all curves, the time needed for dose rate to decrease by twofold is 1 year. Red curve: initial dose rate = 100 µGy/h. Green curve: initial dose rate = 1 µGy/h. Blue curve: initial dose rate = 0.1 µGy/h. Black curve: initial dose rate = 0.01 µGy/h.

Our model-based analysis was able to describe the main patterns of the analyzed plant and rodent data sets using a small number of adjustable parameters (Fig. 2; Tables 1, 2, 3). Predicted radiation responses for various exposure scenarios with constant or exponentially decreasing dose rates, based on best fits of the model to each data set, are shown in Figs. 3 and 4 for plants and Figs. 5 and 6 for rodents.

**Table 3:** Best-fit model parameter values for the plant and rodent data sets.

Parameter	Meaning and units	Best fit value for plant (*A. thaliana*) data			Best fit value for rodent (*C. glareolus*) data		
			95% CIs			95% CIs	
*k*_1_	Cell activation by non-targeted effects (NTE), µGy^−1^	**3.105**	0.026	> 10^2^	**0.323**	0.187	0.625
*c*_3_	Cell deactivation, years^−1^	**12.983**	0.003	> 10^2^	**4.54 × 10**^**–5**^	2.61 × 10^–5^	8.05 × 10^–5^
*k*_*bac*_	Background damage formation, years^−1^	**0.044**	0.021	0.067	**1.07 × 10**^**–6**^	2.79 × 10^–7^	1.86 × 10^–6^
*k*_*TE*_	Damage formation by targeted effects (TE), µGy^−1^	**2.15 × 10**^**–6**^	4.95 × 10^–7^	1.31 × 10^–5^	**3.17 × 10**^**–9**^	< 10^–9^	4.36 × 10^–4^
*k*_*NTE*_	Relationship between NTE activation probability and damage formation, years^−1^	**0.057**	0.023	0.094	**0.025**	0.021	0.030
κ	Damage removal, years^−1^	**0.515**	0.166	1.228	**4.80 × 10**^**–5**^	4.68 × 10^–5^	4.45 × 10^–4^

Best-fit parameter values are indicatd in bold.

These results suggest that NTE rather than TE dominated the responses of both organisms to protracted low-dose-rate irradiation (Figs. 3, 5). In both species, the TE contribution to the radiation response because numerically substantial, relative to NTE, only at dose rates ≥ 10,000 µGy/h. Such high dose rates were present in the plant data set (Table 1), but not in the rodent data set (Table 2). For rodent data, the TE component therefore represents an extrapolation. However, because the dose rate range ≥ 10,000 µGy/h was investigated by many laboratory studies, the importance of TE for intense radiation exposures is expected.

The presumed importance of NTE at lower dose rates in the current analysis is based on the properties of the model used here (described in “Materials and methods” section). The model attributes nonlinear concave radiation response shapes to NTE, and the observed nonlinearities in the analyzed data at low dose rates are fitted by NTE parameters. Model parameters for both NTE and TE, and their uncertainties, are shown in Table 3 for plants and rodents.

Since the model consists of a system on nonlinear differential equations, parameters can affect the fit in combinations (e.g.* k*_1_/*c*_3_, *k*_*bac*_/κ), and the influences of each parameter or combination are not the same. For these reasons, some parameters had much larger uncertainties than others (Table 3). For example, parameter *k*_1_, which represents cell activation by non-targeted effects, was very uncertain in both data sets.

Nevertheless, comparison of best-fit radiation response parameter values for plants and rodents resulted in the following observations: (1) Parameter *c*_3_, which represents cell deactivation from the NTE state, and parameter κ, which represents radiation-induced damage removal over time, were much smaller for rodents, than for plants. (2) Parameter *k*_*NTE*_, which represents the relationship between NTE activation probability and damage formation, was similar across data sets. (3) The TE parameter (*k*_*TE*_) for rodent data is probably unreliable because high dose rates at which TE are likely to become important were not represented in the data set.

These differences in parameter values (particularly *c*_3_ and κ) resulted in qualitative differences in predicted radiation responses for plants are rodents. Specifically, in situations of exponentially decreasing dose rate, radiation-induced embryonic mortality was predicted to continue increasing for considerable time even though dose rate was monotonically decreasing. In Arabidopsis, the predicted time since the start of irradiation needed for embryonic mortality to peak was a few years/generations, and a rapid decline was predicted afterwards (Fig. 4). In voles, the predicted time needed for the effect to peak was much longer than the maximum time in the data set (10 years after the accident, corresponding to ~ 20 generations) (Fig. 6).

In summary, model-based analysis suggested that both Arabidopsis and voles are susceptible to presumably NTE-mediated transgenerational effects such as embryonic mortality, but in voles the NTE state is much more persistent and the damage is removed from the population much more slowly. Consequently, in Arabidopsis the peak yield of radiation-induced damage was predicted to occur within a few years/generations after the start of exposure to an exponentially decreasing dose rate (Fig. 3), whereas in voles the peak damage yield could occur several decades (> 20 generations) after the start of exposure (Fig. 6), when the dose rate was already very low.

## Discussion

Since individual cells in a multicellular organism constantly interact by various forms of signaling, damage caused by ionizing radiation in some cells generates signals that affect the responses of other cells and can induce them to enter into a prolonged stressed state. This state can be transferred even across generations (probably epigenetically)^11, 34^. This phenomenon implies that descendants of severely irradiated individuals can continue to have elevated rates of damage, even if they themselves were exposed much less severely or not at all. The data sets analyzed here^50, 51^ represent likely examples of this phenomenon under environmental—not laboratory—conditions. In particular, the vole data show elevation of embryonic mortality over multiple generations, even though radiation dose rates during this period were decreasing^51^. This pattern is strongly suggestive of non-targeted effects (NTE) such as genomic instability^51^. Our simple mathematical model was able to generate behaviors consistent with this finding by being capable of generating predictions where the radiation effect continues to grow even when the dose rate is reduced dramatically from its initial maximal value.

Radiation-induced NTE were previously detected and studied in *Arabidopsis thaliana*^18, 19, 22^ and in rodents^52–54^, including transgenerational increases in tumorigenesis^55^ and chromosome aberrations^34^. These findings provide indirect support for the plausibility of the conclusions of the current modeling analysis of Arabidopsis and vole embryonic mortality data from the Chernobyl accident region^50, 51^, which suggest a very important role for NTE in responses to protracted low dose rate irradiation in both species. Although Arabidopsis and rodents are phylogenetically very distant and differ strongly in genome size and intrinsic radiosensitivities, both appear to be susceptible to radiation-induced NTE. Transgenerational damage, attributed mainly to NTE by our radiation response model (described in “Materials and methods” section), was particularly persistent in voles, but was removed from the population more rapidly in Arabidopsis.

In voles, this phenomenon resulted in continuously increasing rather than decreasing embryonic mortality over 10 years (~ 20 generations) after the accident, even though radiation dose rates decreased dramatically over this period^51^. In Arabidopsis, the response was maximal a few years/generations after the accident, and decreased afterwards^50^. These qualitative differences in terms of presumed long-term persistence of NTE could be caused by a variety of factors, such as differences in the mode of reproduction between animals (direct production of gametes) and vascular plants (alternation of gametophyte and sporophyte generations).

Both types of radiation response patterns—transiently peaking or persistent transgenerational effects—were decently described by our simple TE + NTE model, with different parameters for the plant and rodent data sets. The NTE-dominant interpretation of the radiation response behaviors at low dose rates is consistent with other studies of these and similar data^46, 51, 56^. However, alternative explanations are also possible, e.g. changes in radiation damage repair efficiency as function of dose rate^50^. The modeling approach and data sets also had important limitations in terms of model simplicity, fitting methodology, and limited data sample size and quality. For example, it is not known which specific NTE mechanisms could be involved in the observed radiation effects on Chernobyl animals and plants. Many environmental variables, such as temperature, humidity, soil type and resource availability, which probably had big effects on the studied organisms, were not available in the analyzed data sets and could not be modeled explicitly, resulting in relatively low R^2^ values for model fits. Despite these limitations, we believe that the results obtained here support the concept of NTE involvement radiation-induced health risks from chronic exposures.

## Supplementary Information

Supplementary Information.
