# Supplementary material for: Quantitative modeling of multigenerational effects of chronic ionizing radiation using targeted and nontargeted effects
Source: Sci Rep. 2021 Feb 26;11:4776. doi: 10.1038/s41598-021-84156-2 (PMC7910614; doi:10.1038/s41598-021-84156-2)
Supplement: Supplementary file 1 — Supplementary Information. [file 41598_2021_84156_MOESM1_ESM.docx]

**Quantitative Modeling of Multigenerational Effects of Chronic Ionizing Radiation Using Targeted and Nontargeted Effects**

Igor Shuryak^1*^, David J. Brenner^1^.

^1^Center for Radiological Research, Columbia University Irving Medical Center, New York, NY, USA

^*^ Corresponding author: Igor Shuryak, M.D., Ph.D.

Center for Radiological Research, Columbia University,

630 West 168^th^ street, VC-11-234/5, New York, NY, 10032

Phone: 212-305-2405; Fax: 212-305-3229

E-mail: is144@cumc.columbia.edu

**Supplementary Methods**

A customized algorithm for fitting the model (Eq. 9 substituted into Eq. 2, in the main text) to the data (Tables 1 and 2, in the main text) was written because model predictions as function of parameter values could not be generated analytically, but needed to be produced by numerically solving a system of differential equations. This excluded the use of many popular nonlinear regression software packages. The iterative fitting procedure consisted of the following steps:

1. Choose initial parameter values (*k_1_*, *c_3_*, *k_bac_*, *k_TE_*, *k_NTE_*, κ) and store them as best values.
2. Evaluate the optimization function *F_opt_* and store it as the best value *F_opt_best_*.
3. Choose new parameter values in the vicinity of the previous best values. This task was implemented by the following procedure (in pseudo-code notation), where β is the generic designation for the selected parameter (*k_1_*, *c_3_*, *k_bac_*, *k_TE_*, *k_NTE_*, or κ), β_best_ is its stored best value, β_r_ is its current randomly-altered value, *U*(0,1) is a uniformly-distributed random number between 0 and 1, *P_big_* is the probability of making a big change to the selected parameter (set to 0.3 after preliminary exploration), *N*(mean, SD) is a normally-distributed random number with the specified mean and standard deviation (SD), β*_best_*__δ_ is the stored magnitude and direction of the parameter change that improved *F_opt_* (starting at 0 for the first iteration), *S_big_* is the size of a big parameter change (set to 1 after preliminary exploration), *i* is the current iteration number, *S_small_* is the size of a small parameter change (set to 0.1 after preliminary exploration), and β_δ_ is the current magnitude and direction of the parameter change:

if *U*(0,1) < *P_big_* then

β_r_ = β_best_×exp[*N*(mean=β*_best__*_δ_, SD=*S_big_*/*i*^1/2^)]

else

β_r_ = β_best_×exp[*N*(mean=0, SD=*S_small_*/*i*^1/2^)]

end if

β_δ_ = ln[β_r_/β_best_]

1. Evaluate *F_opt_* again. If its value (*F_opt_r_*) is better than the stored best value (*F_opt_best_*), update the best parameters to the current ones and update the best function value to the current one. This procedure was implemented as follows:

if *F_opt_r_* > *F_opt_best_* then

*F_opt_best_* = *F_opt_r_*

β_best_ = β_r_

β*_best__*_δ_ = β_δ_

end if

1. Repeat steps 3-4 until convergence criteria (*F_opt_best_* improves by only <10^-4^ over the last 1000 iterations) are satisfied.

The data analyzed here were highly variable even at similar dose rates, which is to be expected for radiation responses under environmental conditions where multiple influential variables cannot be controlled or adequately measured, as could be done in laboratory settings. By starting the optimization procedure at various randomly-chosen initial parameter values, we found that using the sum of squared errors as *F_opt_* frequently resulted in local optima with poor fit quality. To address this issue, we used a customized optimization function *F_opt_* = R^2^×exp[-*w*×RMSE], where R^2^ is the coefficient of determination (square of the Pearson correlation coefficient between *P_mort_* model predictions and observed data values), RMSE is root mean squared error, and *w* is an adjustable parameter. Exploratory calculations showed that maximizing *F_opt_* with *w* = 3 converged to a likely global optimum even when very different initial parameter values were used. For example, in the plant data set, the same best-fit parameter values were eventually found starting from the following initial parameter value combinations: (1) *k_1_* = 3.5×10^-7^ µGy^-1^, *c_3_* = 0.82 years^-1^, *k_bac_* = 0.036 years^-1^, *k_TE_* = 8.1×10^-7^ µGy^-1^, *k_NTE_* = 0.6 years^-1^, κ = 0.24 years^-1^; (2) *k_1_* = 3.0×10^-8^ µGy^-1^, *c_3_* = 0.1 years^-1^, *k_bac_* = 0.006 years^-1^, *k_TE_* = 3.0×10^-6^ µGy^-1^, *k_NTE_* = 0.01 years^-1^, κ = 0.05 years^-1^. Using other options, such as minimization of RMSE alone as the fitting criterion, produced very similar best-fit parameter values.
